# Short-term effects of the strengthening families Program (SFP 10–14) in Brazil: a cluster randomized controlled trial

**DOI:** 10.1186/s13034-024-00748-6

**Published:** 2024-06-06

**Authors:** Zila M. Sanchez, Juliana Y. Valente, Fabiane A. Gubert, Patrícia P. O. Galvão, Hugo Cogo-Moreira, Lidiane N. Rebouças, Miguel Henrique S. dos Santos, Márcia H. S. Melo, Sheila C. Caetano

**Affiliations:** 1 Department of Preventive Medicine, Federal University of São Paulo (UNIFESP), Rua Botucatu, 740 - Vila Clementino, São Paulo, SP 04023-062 Brazil; 2 Department of Nursing, Federal University of Ceará (UFC), Rua Alexandre Baraúna, 1115 - Rodolfo Teófilo, Fortaleza, CE 60430-160 Brazil; 3 Department of Education, Østfold University College, ICT and Learning, Halden, Norway; 4 Department of Psychiatry, Universidade Federal de São Paulo, Rua Major Maragliano, 241 - Prédio Acadêmico - Vila Mariana, São Paulo, Brazil

**Keywords:** Prevention programs, Parenting skills, Drug use, Adolescents, Family, randomized controlled trial

## Abstract

**Introduction:**

This study reports the evaluation of the short-term effects of the Strengthening Families Program (SFP 10–14), adapted as *Famílias Fortes* (Strong Families) in Brazil, on preventing adolescent drug use and improving parenting behaviors.

**Methods:**

A two-arm, parallel cluster randomized controlled trial was conducted in 60 Social Assistance Reference Centers (SARC) from 12 Brazilian municipalities. In each city, the SARC were randomly assigned to the intervention or control group. A total of 805 families participated in the study, each contributing data from one parent or legal guardian and one adolescent totaling 1,610 participants. Data collection occurred before intervention implementation and 6 months after baseline collection. Data were analyzed using multilevel mixed-effects modeling with repeated measure*s* in two different paradigms: *Intention to Treat* (ITT) and *Per protocol* (PP). The study was registered in the Brazilian Ministry of Health Register of Clinical Trials (REBEC), under protocol no. RBR-5hz9g6z.

**Results:**

Considering the ITT paradigm, the program reduced the chance of parents and legal guardians being classified as negligent by 60% (95%CI 0.21; 0.78), increased the use of nonviolent discipline by caregivers (Coef 0.33, 95%CI 0.01; 0.64) and decreased the chance of adults exposing adolescents to their drunken episodes by 80% (95%CI 0.06; 0.54). No program effects were observed on outcomes related to adolescent drug use. Similar results were found for the PP paradigm.

**Conclusion:**

The positive effects on family outcomes suggest preventive potential of the program among the Brazilian population. Long-term evaluations are necessary to verify if the program can also achieve the drug use reduction goals not observed in the short term.

## Introduction

Alcohol and other drug use during adolescence are important markers for the global burden of disease in adulthood [1], and are among the leading causes of disability-adjusted life years lost among youth [2]. According to the 2019 PeNSE (Brazilian National School Health Survey), 22.6% of Brazilian students from 13 to 17 years of age had experimented with cigarettes, while 63.3% had experimented with alcohol [3]. Additionally, 13.0% of these students had used an illicit drug at some point in their lives, with recent marijuana use (30 days prior to the survey) reported by 5.3% [3]. Public policies aimed at reducing drug use [4] is essential in Brazil, especially considering that one in every five Brazilian adolescents reported getting drunk before age 13 [3].

Family-based drug use prevention programs have been implemented worldwide to decrease risk factors and increase protection for early alcohol and other drugs use [5]. These programs focus on developing and strengthening parenting skills, which act as mediators in reducing drug use by children and adolescents, and have shown the highest degree of evidence of effectiveness among interventions [6].

Strengthening Families Program (SFP) is an intervention aimed at preventing adolescent drug use and other behavioral problems by promoting parenting skills and strengthening the family bond [7]. According to its logic model [8], short-term effects on parenting skills, adolescents’ social and personal skills, and family bonds would lead to long-term outcomes, such as reduced drug use, conduct disorders, school dropout, and criminal activities. The program’s syllabus is grounded on the following social and psychological theories: (1) Family Systems Theory [9], which addresses the importance of individuation and autonomy in family relations for development of better mental health; (2) Social Learning Theory [10], which suggests that behavior can be modeled by observing other individuals’ behavior; (3) Kumpfer’s Resilience Theory [11], which emphasizes the importance of goals and purpose in life for resilience development; and (4) Reinforcement and Punishment Theory, which suggests that positive reinforcement by parents and legal guardians can increase desired behaviors, and punishment or neglect can increase undesired behaviors [12].

SFP for children and adolescents aged 10 to 14 years (SFP10-14) has undergone several effectiveness evaluations in Europe, Latin America, and the United States. Regarding the program’s primary outcomes, consistent positive effects were reported by studies conducted primarily in the United States, which showed late initiation and reduced frequency of alcohol and other drugs use [13–16]. Conversely, some European and Latin American studies found null effects on drug use prevention [17–20].

Regarding family outcomes, North American and Latin American studies have shown improvement in family cohesion and supervision [21]; increased display of affection towards the child, father’s communication and involvement with the child, family functionality, supervision of norm compliance (1); decreased aggressive behavior in family interactions [22]; resilience, involvement, family conflicts, positive parenting [23] and reduced violent parenting practices [24], despite contradictory effects for adolescent “problem-solving skills” and “parent-youth hostility” [25].

Considering the mostly positive SFP evidence found in other countries, its version developed by Oxford Brookes University, United Kingdom [26], was adapted to the Brazilian context where it was renamed *Famílias Fortes* and implemented in “Social Assistance Reference Centers” (SARC). Between 2013 and 2015, the program underwent cultural adaptation to the Brazilian population attending SARC in six sub-districts of Distrito Federal [27, 28]. The main goal was to maintain the core components in each lesson while adapting language, slang and situations described. Some examples of the changes introduced were the replacement of “rugby” and “basketball” with “football” to better align with the Brazilian context. Additionally, certain written activities were modified to oral formats to accommodate participants with limited literacy skills while preserving the program’s integrity [28].

From 2013 to 2019, the program was implemented by the Ministry of Health and monitored via evaluation with a “pre-experimental” design [29]. In 2019, the program began to be implemented as a public policy under the responsibility of the Ministry of Women, Family and Human Rights (MMFDH) by means of a public call for municipalities. MMFDH considered it essential to assess the program based on a gold standard study of effectiveness to guide decision-makers on whether to maintain its diffusion or make adaptations to this public policy. As the effects of interventions may not be replicated in different cultures [30], conducting randomized controlled trials (RCT) is essential to evaluate the effectiveness of the *Famílias Fortes* program in Brazil.

This study investigates whether the *Famílias Fortes* program, in the short term, is effective in preventing lifetime drug use and increasing risk perception by adolescents and in improving parenting behaviors that mediate adolescent drug use prevention (such as family skills, reduced family violence, and reduced parental exposure to drug use), measured along a 6-month follow-up.

## Method

Effectiveness of the *Famílias Fortes* program was evaluated by a two-arm cluster randomized controlled trial (RCT) conducted in 60 Social Assistance Reference Centers (SARC) and similar municipal services, hereafter referred to globally as “social services,” from 12^1^ municipalities designated by the Federal Government. These SARC are public centers responsible for delivering social programs, benefits and projects to low-income families and individuals in the community, including registering and monitoring the families benefited by Bolsa Família, a a social welfare program provided by the government to extremely low-income families. The existing SARCs were randomized in each city between intervention and control groups. Each SARC invited 15 families to participate in the study from the list of attended families and considering the inclusion criteria (have at least one child/adolescent between 10 and 14 years old; adult and teenagers living in the same house; availability to attend the 7 program meetings; live up to 1 km away from the social centers) and exclusion criteria (families with very high needs or challenges such as drug addiction and family breakdown). If the SARCs had more than 15 families interested, the first to volunteer were included in the study.

The families (made up of parents or legal guardians and child/adolescent) in the intervention group attended the *Famílias Fortes* program for seven weeks in person, while those from the control group were put on a waiting list to participate in the program after the end of the study. Data collection occurred at two time points: before the intervention (November/December 2021), and 6 months after implementation (May/June 2022).

Families answered self-completed, anonymous, virtual questionnaires via Android smartphone app or Internet link provided by the researchers. Parent and adolescent data were integrated through linkage considering the family confidential code. For any family code typing errors, the Levenshtein algorithm was used to pair the subjects, as described in previous studies [31, 32].

### Intervention

The *Famílias Fortes* program (PFF-BR 10–14) is the Brazilian adaptation of the Strengthening Families Programme (SFP-UK 10–14), developed in the United Kingdom by the Oxford Brookes University [26]. PFF-BR 10–14 consists of seven consecutive weekly 2-hour in-person meetings. Caregivers and children meet separately in the first hour and spend the second hour together in family activities. All meetings include debates, games, and interactive activities and a snack is offered at the end. Some sessions have the support of videos depicting situations of daily family life. The themes of these meetings are: (1) support goals and dreams; (2) admire family members; (3) family moments; (4) understanding family values; (5) strengthen family communication; (6) family and pressure from friends; and (7) putting it all together. Highly structured, the program is guided by the instructor, caregiver, and youth manual, all available at https://www.gov.br/mdh/pt-br/navegue-por-temas/familia/familias-fortes-1.

The professionals who delivered the program (called implementers) were typically SARC employees, such as psychologists and social workers, who had been previously serving the participating families and had undergone specific training for program implementation. Program methodology training for implementers was supervised and monitored by the MMFDH team. It was conducted online via a digital platform developed specifically for distance learning courses, the training was configured as a 25-hour course divided into 3 modules: (1) Introduction and theoretical grounds of the *Famílias Fortes*; (2) Training to conduct the *Famílias Fortes* meetings, and (3) Practices for the follow-up and closing of the *Famílias Fortes*.

Importantly, the program’s cultural adaptation was carried out in 2014 and 2015 by the Federal University of Brasilia [27]. In this process, European everyday examples were replaced by activities typical to Brazilian everyday life. The branding, vocabulary, and presentation format of some activities were also adapted without changes in content and core elements. Evaluations of this culturally adapted version [33, 34] concluded that the program was sufficiently attractive, culturally relevant, with acceptable goals, and compatible with the needs of Brazilian families in vulnerable contexts (the target audience of the SARC).

### Instruments and measures

Adolescents and caregivers each answered a self-completed, virtual, anonymous questionnaire via an Android smartphone app or Internet link made available by the researchers during data collection, without any participation of the implementers. The two questionnaires, one for parents/guardians and the other for adolescents, were built based on international instruments developed for evaluating the Strengthening Families Program (SFP 10–14) [35] in combination with instruments previously employed in effect evaluation studies of drug use prevention programs in Brazil [36, 37]. Such instruments were extracted from questionnaires widely used in several national and international studies on drugs such as the World Health Organization questionnaire used by the Brazilian Center for Drug Information (CEBRID) [38] and that used by the Substance Abuse and Mental Health Services Administration [39].

In this study, parental outcomes (family violence, parenting styles, and children’s exposure to parental drug use) and adolescent outcomes (perceived drug risk and lifetime drug use) were assessed both at baseline and at 6-month follow-up.

### Parental outcomes

Family violence was assessed using the WorldSAFE Core Questionnaire scale [40] translated and validated into Brazilian Portuguese [41]. Questionnaire questions are grouped into 5 subscales: Nonviolent Discipline, Moderate Verbal Discipline, Severe Verbal Discipline, Moderate Physical Discipline, and Severe Physical Discipline. The instrument asks how often caregivers have used specific disciplinary tactics, with responses rated on a 3-point scale: never, 1–2 times, and ≥ 3 times in the previous three months. The Moderate Verbal Discipline and Severe Verbal Discipline subscales were grouped together and its corresponding score ranged from 0 to 16, in which the higher the value the higher the degree of verbal discipline imposed. The Moderate Physical Discipline and Severe Physical Discipline subscales were also grouped together and the score ranged from 0 to 24, in which the higher the value the greater the degree of physical discipline effected. Nonviolent Discipline score ranged from 0 to 10, and the higher the value, the greater the degree of nonviolent discipline employed.

Parenting styles were evaluated by the Scale of Parental Demands and Responsiveness [42], translated and validated into Portuguese [43]. Each instrument item (six items composing the demanding dimension; and ten items the responsive dimension) is assessed by a 3-point Likert scale, where values closer to three indicate greater perceived demand and responsiveness (ranging from 0 to 12 and 0 to 20, respectively). Parenting style dimensions were established using the median split procedure following the methodology proposed by previous studies [42–44]. Caregivers who scored at or above the median for demandingness or responsiveness were classified as high in demandingness or responsiveness, whereas caregivers with scores at or below the median were classified as low in demandingness or responsiveness. Parenting styles were organized into four categories combining these two factors: authoritative (scoring high on demandingness or responsiveness), authoritarian (scoring high on demandingness and low on responsiveness), indulgent (scoring low on demandingness and high on responsiveness), and negligent (scoring low on demandingness and responsiveness). Prior psychometric evaluation of the scale in a sample of Brazilian adolescents showed good fit: χ2 = 1518.249, *p* < 0.001, RMSEA = 0.050, CFI = 0.940, TLI = 0.929, WRMR = 2.377 [45].

Child exposure to parental drug use was measured by means of a block of questions that assessed the degree to which caregivers exposed their child to drug use (alcohol, cigarette, marijuana, and cocaine), such as “Do you get drunk near your child?,” and similarly to social drinking, smoking and use of illegal drugs, whose response options could be “never, sometimes, and always.”

### Adolescent outcomes

Adolescent drug use was assessed as lifetime drug use (alcohol, cigarette, marijuana, inhalants, and binge drinking) and risk perception (alcohol, cigarette, and marijuana) measured by questions drawn from the CEBRID [38] and SAMHSA [39] questionnaires. Lifetime drug use (yes X no) was assessed through questions such as “Have you ever tried any alcoholic beverages?” Perceived risk was measured by one question for each drug with Likert-type response options ranging from no risk to high risk, such as “How risky is it for someone your age to smoke cigarettes once or twice?”

Covariates included age, gender, race, and socioeconomic status (SES) of the caregivers and adolescents. SES was assessed using the Brazilian Association of Research Enterprises (ABEP) scale [46], which considers the schooling of the head of the household and goods and services used by the household. ABEP score ranged from 1 to 100 points, with categories ranging from highest to lowest according to the cut-off points established in literature: high (45–100), medium [29–44], medium-low [17–28] and low (0–16) [46].

### Data analysis

Program effectiveness analysis was performed according to two paradigms: *Intention to Treat (ITT)* and *Per Protocol (PP).* The ITT model considered all families who participated in the study regardless of number of meetings attended and whether or not they answered the questionnaire at follow-up. The PP model, in turn, considered only those families who had participated in at least five program sessions or more and who answered the baseline and 6-month follow-up questionnaires, aiming to ensure program evaluation among people who joined it, similar to another study [21]. Attending at least five sections was considered the protocol for MMFDH dissemination and not an arbitrary decision of the researchers.

Considering the longitudinal hierarchical structure of the data, a multilevel mixed-effects modeling with repeated measures approach was adopted. Changes in intervention outcomes use over time was evaluated by three-level mixed-effects models (level 1: repeated time observations nested within the subject; level 2: subject clustered within SARC; level 3: SARC). Models were calculated using SARC and subjects as a random effect, and explanatory variables (experimental group, time of assessment, the interaction between group and time), controlling variables (gender, age, SES), and study outcomes as fixed effects. Unadjusted estimations were obtained when considering the effect of only one independent (predictor) variable and adjusted estimations were controlled for covariates (sex, age, and socioeconomic status). Standard errors allow for intragroup correlation, that is, the observations are independent across groups (clusters) but not necessarily within groups. All models were fitted with STATA 17 program generalized linear mixed models (GLMM).

Mixed-effects models are powerful tools for analyzing cluster randomized trials that handle two sources of non-independence: the clustered observations and the repeated measures within each subject over time [47]. Additionally, mixed-effects models deal with missing data using maximum-likelihood estimation analyzing all available outcome data, regardless of whether an individual has complete data, making these models consistent with an ITT analysis [48]. In our sample, we assumed the missing data mechanism as missing at random (MAR), that is, when the probability of missing data on a variable is related to other variables measured in the model rather than the variable with missing values itself. Missing data stemmed mostly from failure to answer the follow-up assessment questionnaire and not because single items remained unanswered or due to dropping out. Mixed model for repeated measures under MAR assumption produces valid and unbiased estimations and additional methods for handling missing data, such as multiple imputation, are generally unecessary [47]. For attrition analysis, we compared families whose data from the two time-points had been matched with families who answered only the baseline questionnaire.

## Results

### Participants’ characteristics

**Fig. 1:**
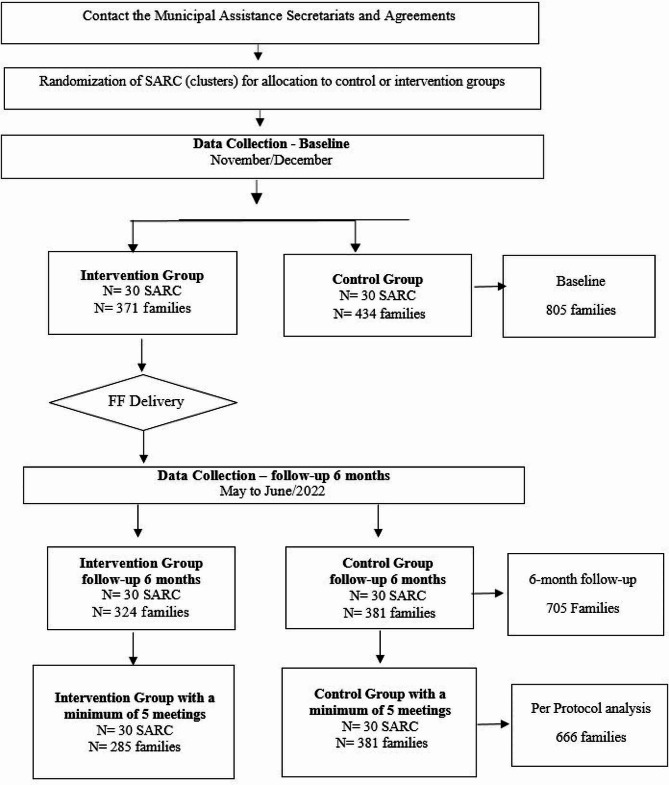
Flowchart of the randomized controlled trial of the *Famílias Fortes* program

Data were collected from 805 families at baseline, 371 in the intervention group and 434 in the control group (Fig. 1). At the 6-month follow-up, 12.4% of participants were lost over time, thus valid data were collected from 705 families, 324 in the intervention group and 381 in the control group. Of the 324 families in the intervention group, 89% received five or more intervention lessons, that is, they attended at least five of the seven program meetings.

The sample of adolescents consisted of 51.5% girls with a mean age of 12.6 years and mainly from the lower social status (73.5%). Caregivers were mainly adult females (91.8%) with a mean age of 39.5 (± 8.11). At baseline, the drug most used by adolescents was alcohol (13.3%) and marijuana was the substance with the highest perceived risk (75.3%). Most parents/guardians reported not exposing their children to alcohol and other drugs. Authoritative (38.3%) was the most prevalent parenting style, followed by negligent (29.1%). As for family violence, the control group showed higher scores for nonviolent discipline (3.3 ± 1.6 and 3.1 ± 1.6, respectively) and more total violence compared with the intervention group (25.5 ± 2.6 and 24.8 ± 3.7, respectively) (Table 1).

**Table 1:** Sociodemographic, family and drug consumption characteristics of the total sample of the baseline of the study that evaluated the effect of the Forte Families program (*N* = 805)

	Total *N* = 805		Control group *N* = 434		Intervention group *N* = 371		*p*-value*
	*N*	%	*N*	%	*N*	%	
Gender (adolescents)							
Male	384	47.7	204	47.0	180	48.5	0.764
Female	415	51.5	226	52.1	189	51.0	
Others	6	0.8	4	0.9	2	0.5	
Age (adolescents)– (mean ± SD)	805	12.6 ± 1.24	434	12.6 ± 1.25	371	12.6 ± 1.24	0.890
Gender (adults)							
Male	63	7.8	33	7.6	30	8.1	0.747
Female	739	91.8	400	92.2	339	91.4	
Others	3	0.4	1	0.2	2	0.5	
Age (adults)– (mean ± SD)	805	39.5 ± 8.11	434	39.1 ± 8.00	371	39.8 ± 8.28	0.228
Socioeconomic status (adults)							
High	7	0.9	3	0.7	4	1.1	0.001
Medium	31	3.9	8	1.8	23	6.2	
Medium-low	175	21.7	83	19.1	92	24.8	
low	592	73.5	340	78.4	252	67.9	
Lifetime use of drugs (adolescents)							
Alcohol	106	13.3	56	13.1	50	13.6	0.857
Binge drinking	32	4.1	17	4.0	15	4.1	0.948
Tobacco	19	2.4	13	3.0	6	1.6	0.189
Inhalants	60	7.6	33	7.8	27	7.3	0.812
Risk perception of drug use (adolescents)							
Alcohol							
No risk	61	7.7	29	6.8	32	8.7	0.293
Light risk	87	11.0	41	9.7	46	12.5	
Moderate risk	203	25.6	117	27.5	86	23.4	
High risk	441	55.7	238	56.0	203	55.4	
Tobacco							
No risk	53	6.7	28	6.6	25	6.8	0.689
Light risk	65	8.2	31	7.3	34	9.2	
Moderate risk	193	24.3	101	23.7	92	25.0	
High risk	483	60.8	266	62.4	217	59.0	
Marihuana							
No risk	49	6.2	25	5.9	24	6.5	0.775
Light Rrisk	41	5.1	19	4.5	22	6.0	
Moderate risk	106	13.4	57	13.4	49	13.3	
High risk	597	75.3	324	76.2	273	74.2	
Exposure of the child to drug use (adults)							
Social drinking							
Never	595	74.6	325	75.4	270	73.6	0.086
Sometimes	168	21.1	82	19.0	86	23.4	
Always	35	4.3	24	5.6	11	3.0	
Get drunk							
Never	742	93.5	410	95.4	332	91.2	0.002
Sometimes	45	5.7	14	3.3	31	8.5	
Always	7	0.8	6	1.3	1	0.3	
Smoke							
Never	708	89.5	386	90.0	322	89.0	0.684
Sometimes	47	5.9	26	6.1	21	5.8	
Always	36	4.6	17	3.9	19	5.2	
Parenting style (adults)							
Authoritative	290	38.3	169	41.4	121	34.7	0.063
Authoritarian	124	16.4	71	17.4	53	15.2	
Indulgent	123	16.2	65	15.9	58	16.6	
Negligent	220	29.1	103	25.3	117	33.5	
Family Violence (adults)							
Nonviolent discipline (Average ± SD)	805	3.19 ± 1.57	434	3.29 ± 1.58	371	3.06 ± 1.56	0.038
Total diolence (Average ± SD)	805	25.16 ± 3.16	434	25.46 ± 2.56	371	24.82 ± 3.71	0.004
Verbal discipline (Average ± SD)	805	7.35 ± 1.28	434	7.44 ± 1.13	371	7.26 ± 1.42	0.044
Physical discipline (Average ± SD)	805	17.25 ± 1.95	434	17.40 ± 1.45	371	17.07 ± 2.39	0.016

*We conducted a chi-square test for categorical variables and a t-test for continuous variables

Table 2 summarizes the descriptive data for the study variables of interest (drug use, risk of drug use, parental drug use exposure, parenting styles, and family violence) concerning the control and intervention groups. Lifetime drug use increased over time in both groups. Regarding risk of drug use, the perception of high risk increased between the two time-points for the three drugs (alcohol, cigarettes, and marijuana) and for both groups. As for parenting styles, both groups showed an increase in the percentage of the authoritative style between the two time-points, whereas the intervention group had a reduction in the negligent style. Both groups presented descriptive differences between the time points for family violence scores. For example, we observed a statistically significant difference in the nonviolent discipline score of 0.30 in the control group and 0.45 in the intervention group.

**Table 2:** Distribution of variables related to lifetime drug use, risk of drug use, exposure to drug use, parenting styles, and family violence concerning the control and intervention group (*n* = 805)

	Control						Intervention					
	Baseline		Follow-up		Change (Baseline-Follow-up)		Baseline		Follow-up		Change (Baseline-Follow-up)	
	n	%	n	%	%	95%CI	n	%	n	%	%	95%CI
Lifetime drug use by adolescents												
Alcohol	56	13.11	85	22.19	8.30	5.9; 10.6	51	13.82	76	22.89	7.84	5.30;10.38
Binge drinking	17	4.03	31	8.14	4.17	1.50; 6.84	15	4.12	20	6.13	1.93	− 0.68;4.53
Tobacco	13	3.04	20	5.22	1.78	0.06; 0.03	6	1.62	10	3.08	1.18	− 0.41;2.76
Inhalants	33	7.76	33	8.62	1.72	− 1.85; 5.31	27	7.32	28	8.64	2.98	− 0.90;6.88
Risk perception of drug use by adolescents												
Alcohol												
No risk	29	6.82	15	3.94	− 2,5	− 4.0; − 1.0	32	8.72	16	4.95	− 2.4	− 4.1; − 0.6
Light risk	41	9.65	31	8.14	− 3.0	− 4.8; − 1.2	46	12.53	232	9.91	− 2.7	− 4.7; − 0.7
Moderate risk	117	27.53	84	22.05	− 3.9	− 6.3; − 1.6	86	23.43	73	22.60	− 3.1	− 5.4; − 0.8
High risk	238	56.00	251	65.88	9.5	4.1; 14.9	203	55.31	202	62.54	8.2	2.3; 14.1
Tobacco												
No risk	28	6.57	13	3.41	− 1.8	− 3.2; − 0.4	25	6.79	16	4.95	− 2.2	− 3.9; − 0.6
Light risk	31	7.28	27	7.09	− 1.9	− 3.4; − 0.4	34	9.24	22	6.81	− 2.3	− 3.9; − 0.6
Moderate risk	101	23.71	76	19.95	− 3.4	− 6.0; − 0.7	92	25.00	66	20.43	− 3.7	− 6.4; − 1.0
High risk	266	62.44	265	69.55	7.1	1.7; 12.4	217	58.97	219	67.80	8.2	2.3; 14.1
Marihuana												
No risk	25	5.88	15	3.94	− 2.3	− 3.84; − 0.72	24	6.52	19	5.88	− 1.06	− 2.96; 0.84
Light risk	19	4.47	15	3.94	− 1.6	− 6.67; − 0.49	22	5.98	15	4.64	− 0.7	− 1.87; 0.54
Moderate risk	57	13.41	33	8.66	− 3.1	− 5.24; − 1.03	49	13.32	40	12.38	− 1.2	− 3.35; 0.96
High risk	324	76.24	318	83.46	7.0	2.39; 11.60	273	74.18	249	77.09	2.9	− 2.31; 8.16
Exposure of the child to drug use												
Social drinking												
Never	325	75.41	298	78.63	1.7	− 1.75; 5.11	270	73.57	249	77.09	3.0	− 0.59; 6.51
Sometimes	82	19.03	57	15.04	− 1.2	− 3.55; 1.22	86	23.43	65	20.12	− 0.2	− 4.54; 0.42
Always	24	5.57	24	6.33	− 0.5	− 1.57; 0.54	11	3.00	9	2.79	− 0.9	− 2.00; 0.19
Get drunk												
Never	410	95.35	355	93.18	− 1.8	− 4.42; 0.84	332	91.21	307	95.94	4.3	1.50; 7.09
Sometimes	14	3.26	19	4.99	1.4	− 0.68; 3.55	31	8.52	13	4.06	− 3.4	− 5.71; − 1.15
Always	6	1.40	7	1.84	0.4	− 0.20; 0.91	1	0.27	0	0.00	− 0.9	− 1.56; − 0.16
Smoke												
Never	386	89.98	336	88.65	− 2.3	− 4.78; − 0.14	322	88.95	286	89.10	− 0.9	− 3.50; 1.70
Sometimes	26	6.06	23	6.07	0.8	− 0.79; 2.31	21	5.80	14	4.36	0.3	− 0.68; 1.21
Always	17	3.96	20	5.28	1.6	− 0.22; 3.34	19	5.25	21	6.54	0.6	− 1.24; 2.51
Parenting style												
Authoritative	169	41.42	163	43.47	2.67	− 3.5; 8.9	121	34.67	148	47.13	13.07	6.4; 19.8
Authoritarian	71	17.40	62	16.53	− 0.5	− 5.1; 4.1	53	15.19	40	12.74	− 2.3	− 7.0; 2.4
Indulgent	65	15.93	46	12.27	− 3.1	− 7.5; 1.3	58	16.62	43	13.69	− 2.5	− 7.4; 2.4
Negligent	103	25.25	104	27.73	0.9	− 3.8; 5.7	117	33.52	83	26.43	− 8.3	− 13.6; − 2.9

	Mean ± SD	Mean ± SD	Change	95%CI	Mean ± SD	Mean ± SD	Change	95%CI				
Family violence												
Total violence	25.46 ± 2.56	25.81 ± 2.60	0.36	0.07; 0.65	24.82 ± 3.71	25.60 ± 3.14	0.77	0.31; 1.26				
Verbal discipline	7.38 ± 1.30	6.77 ± 0.77	0.65	− 0.76; − 0.55	7.26 ± 1.42	6.79 ± 0.75	0.48	− 0,63; − 0.32				
Physical discipline	17.40 ± 1.45	17.52 ± 1.45	0.13	− 0.05; 0.31	17.07 ± 2.38	17.37 ± 2.01	0.30	− 0.01; 0.61				

n.e. not estimable

### Intervention effect

Regarding the program’s impact, the statistically significant outcomes, as indicated by the *Intention to Treat* and *Per Protocol* models (Table 3), encompassed the following factors: child exposure to parental drunkenness, parenting styles, and nonviolent discipline. Caregivers who participated in the program reduced their child’s exposure to episodes of drunkenness by 88% in the ITT analysis (95%CI 0.04; 0.41) and by 79% in the PP analysis (95%CI -0.30; 0.52) compared with the control group. Results from the multinomial regression showed an effect of the intervention in modifying parenting styles, indicating a 60% lower odds of caregivers being negligent (versus authoritative) in the intervention group compared with the control group in the two models assessed (OR_ITT_ = 0.40, 95%CI 0.21; 0.78 and OR_pp_ = 0.39, 95%CI 0.19; 0.79). As for nonviolent discipline, caregivers who participated in the program reported increasing this practice by 0.32 (95%CI 0.01; 0.63) and 0.35 (95%CI 0.01; 0.70) points compared with the control group, considering the ITT and the PP models, respectively. The program failed to be effective in reducing lifetime use of any of the drugs surveyed, perceived risk of drug use among adolescents, child exposure to alcohol, tobacco, and illicit drug use, and caregivers physical and verbal violent disciplinary actions.

**Table 3:** Unadjusted and adjusted multilevel logistic and linear models that evaluated the effect of the Strong Families program, according to the Intent-to-Treat (ITT) and Per protocol (PP) paradigms

	Intent-to-treat (805 families)						Per protocol* (666 families)					
	Unadjusted multilevel model			Adjusted multilevel model*			Unadjusted multilevel model			Adjusted multilevel model**		
	OR	95%CI	*p*-value	AOR	95%CI	*p*-value	OR	95%CI	*p*-value	AOR	95%CI	*p*-value
Lifetime drug use by adolescents												
Alcohol	0.87	0.18; 4.14	0.861	0.53	0.11; 2.56	0.426	1.16	0.20; 6.73	0.868	1.06	0.20; 5.58	0.945
Binge drinking	0.42	0.08; 2.05	0.284	0.21	0.02; 1.80	0.155	0.36	0.20; 6.73	0.283	0.42	0.07; 2.39	0.327
Tobacco	0.93	0.12; 7.51	0.946	0.91	0.07; 12.28	0.945	1.06	0.05; 20.83	0.970	1.06	0.06;19.67	0.971
Inhalants	1.41	0.32; 6.23	0.647	1.30	0.32; 5.20	0.712	1.33	0.25; 7.17	0.741	1.38	0.24; 8.01	0.717
Risk perception of drug use by adolescents												
Alcohol	0.92	0.59; 1.44	0.720	1.02	0.31; 3.35	0.973	0.95	0.59; 1.52	0.835	0.96	0.60; 1.54	0.860
Tobacco	1.05	0.66; 1.68	0.822	1.66	0.20; 2.17	0.497	1.01	0.62; 1.66	0.958	1.01	0.62; 1.66	0.964
Marihuana	0.69	0.40; 1.20	0.191	0.68	0.21; 2.24	0.531	0.74	0.42; 1.33	0.320	0.70	0.39; 1.27	0.240
Exposure of the child to drug use												
Social drinking	0.84	0.42; 1.65	0.605	0.90	0.42; 1.90	0.777	0.71	0.34; 1.46	0.349	0.71	0.34; 1.45	0.342
Get drunk	0.17	0.06; 0.54	0.003	0.12	0.04; 0.41	0.001	0.21	0.06; 0.71	0.012	0.21	0.06; 0.71	0.012
Smoke	0.66	0.23; 1.88	0.440	0.57	0.15; 2.18	0.412	0.62	0.21; 1.86	0.397	0.66	0.22; 1.95	0.451
Parenting style***												
Authoritative	Ref			Ref			Ref			Ref		
Authoritarian	0.63	0.32; 1.28	0.203	0.63	0.32; 1.28	0.203	0.58	0.27; 1.26	0.168	0.54	0.25; 1.19	0.126
Indulgent	0.80	0.40; 1.63	0.546	0.80	0.40; 1.63	0.546	0.69	0.32; 1.49	0.343	0.67	0.31; 1.47	0.321
Negligent	0.40	0.21; 0.78	0.007	0.40	0.21; 0.78	0.007	0.40	0.20; 0.80	0.009	0.39	0.19; 0.79	0.009

	Coef	95%CI	p-value	Coef	95%CI	p-value	Coef	95%CI	p-value	Coef	95%CI	p-value
Family violence												
Non-violent discipline	0.32	0.01; 0.63	0.045	0.32	0.01; 0.63	0.044	0.33	-0.01; 0.68	0.056	0.35	0.01; 0.70	0.049
Total violence	0.41	-0.20; 1.02	0.186	0.41	-0.20; 1.01	0.189	0.31	-0.33; 0.95	0.346	0.33	-0.31; 0.98	0.309
Verbal discipline	0.19	-0.01; 0.39	0.666	0.18	-0.02; 0.37	0.076	0.11	-0.09; 0.31	0.302	0.12	-0.08; 0.32	0.246
Physical discipline	0.17	-0.22; 0.56	0.389	0.17	-0.22; 0.56	0.389	0.10	-0.30; 0.51	0.628	0.11	-0.30; 0.52	0.596

n.e. not estimable

*We considered only those families who had participated in at least 5 program sessions or more and who responded to the baseline and 6-month follow-up questionnaires

**Adjusted for sex, age, and socioeconomic status. The reference is the control group

***We conducted a multinomial regression for parenting style outcome and authoritative is the reference

### Attrition

Attrition was not differential by intervention condition. When comparing families who were lost at the 6-month follow-up and those who could be located, the attrition analysis showed no statistically significant difference for most sociodemographic variables and the surveyed outcomes measured at baseline. Regarding sociodemographic variables, families lost at the 6-month follow-up were slightly younger (12.2%) compared with those who completed the follow-up questionnaire (12.7%). As for outcomes, inhalant use at baseline was more common among families who did not complete the questionnaire at follow-up (12.6%) compared with those who did (6.9%). A statistically significant difference was observed in parenting styles: lost families reported that their caregivers were more forgiving (29%) and less negligent (11%) compared with followed families (14.3% and 31.8%, respectively). Further details of these analyses are shown in Table 4.

**Table 4:** Distribution of sociodemographic variables and outcomes among participants in the cluster-randomized controlled trial of the evaluation of the *Famílias Fortes* Program according to participants who answered the questionnaire at the 6-month follow-up and those who were lost. Attrition analysis for covariates (*N* = 805)

Sociodemographic variables and drug use	Losses**		Follow-up*		pª
	(*N* = 100)		(*N* = 705)		
	*N*	%	*N*	%	
Group					
Control	53	53.00	381	54.04	0.845
Intervention	47	47.00	324	45.96	
Gender (adolescents)					
Male	47	47.47	336	47.86	0.646
Female	52	52.53	360	51.28	
Others	0	0.00	6	0.85	
Gender (adults)					
Male	11	11.00	52	7.39	0.371
Female	89	89.00	649	92.19	
Others	0	0.00	3	0.43	
Age (adolescents)– (mean ± DP)	12.2 ± 1.28		12.7 ± 1.23		< 0.001
Socioeconomic status					
High	2	2.04	5	0.72	0.322
Medium	6	6.12	25	3.58	
Medium-low	19	19.39	156	22.35	
Low	71	72.45	512	73.35	
Lifetime use of drugs by adolescents					
Alcohol	10/97	10.31	96/699	13.73	0.352
Binge drinking	5/95	5.26	27/691	3.91	0.531
Tobacco	4/96	4.17	15/701	2.14	0.222
Inhalants	12/95	12.63	48/699	6.87	0.050
Risk perception of drug use (adolescents)					
Alcohol					
No risk	10	10.53	51	7.32	0.279
Light risk	7	7.37	80	11.48	
Moderate risk	20	21.05	183	26.26	
High risk	58	61.05	383	54.95	
Tobacco					
No risk	6	6.32	47	6.72	0.602
Light risk	11	11.58	54	7.73	
Moderate risk	24	25.26	169	24.18	
High risk	54	56.84	429	61.37	
Marihuana					
No risk	5	5.26	44	6.30	0.457
Light risk	5	5.26	36	5.16	
Moderate risk	8	8.42	98	14.04	
High risk	77	81.05	520	74.50	
Exposure of the child to drug use					
Social drinking					
Never	73	73.74	522	74.68	0.944
Sometimes	22	22.22	146	20.89	
Always	4	4.04	31	4.43	
Get Drunk					
Never	93	94.90	649	93.25	0.585
Sometimes	5	5.10	40	5.75	
Always	0	0.00	7	1.01	
Smoke					
Never	84	85.71	624	90.04	0.424
Sometimes	8	8.16	39	5.63	
Always	6	6.12	30	4.33	
Parenting style					
Authoritative	42	42.00	248	37.75	< 0.001
Authoritarian	18	18.00	106	16.13	
Indulgent	29	29.00	94	14.31	
Negligent	11	11.00	209	31.81	
Family violence					
Nonviolent discipline (mean ± SD)	3.2 ± 1.52		3.2 ± 1.59		0.858
Total violence (mean ± SD)	25.1 ± 3.24		25.2 ± 3.15		0.912
Verbal discipline (mean ± SD)	1.7 ± 0.52		1.6 ± 0.55		0.435
Physical discipline (mean ± SD)	10.3 ± 1.57		10.3 ± 1.58		0.888

*Cases that responded to the questionnaire at baseline and at the 6-month follow-up

**Cases that answered the questionnaire only at the beginning

^a^Chi-Square test

## Discussion

Since a 6-month follow-up period may be insufficient to identify changes in drug use patterns among 12-year-olds, we directed our study toward examining the mediators of program effects. *Famílias Fortes* showed reduced negligent parenting style, increased nonviolent disciplinary practices, and decreased adolescent exposure to episodes of parental drunkenness. The program showed no short-term effect on the prevalence of lifetime alcohol and other drugs use or even increased perceived risk of these substances among adolescents. These findings are similar for the PP and ITT paradigms.

Our most robust finding showed a significant decrease in negligent parenting style in the group attending the *Famílias Fortes* program compared with the control group. Negligent parenting is characterized by low rates of demandingness and responsiveness in parental practices [49], i.e., parents offer low reception and emotional support to their children, in addition to low monitoring and supervision of daily activities and behaviors, such as knowing where and with whom their child is [43]. The scientific literature consensually affirms that responsive and demanding parenting practices are predictors of healthier adolescent development. Studies have shown that these parenting skills are associated with drug use prevention [45] and that children of negligent parents are at higher risk for early involvement with these substances [50]. Previous evaluations on the effect of SFP on family outcomes have also reported results similar to ours, although we could not find any articles that examined parental outcomes using the same variables (parenting styles). A US study developed in public schools found an effect of the program on improving family supervision six months after the intervention [3]. A quasi-experimental study on the Colombian adaptation of the program showed an increase in parenting skills such as expressions of affection, communication, supervision, and parental involvement in the lives of children at 12 and 18 months of follow-up [51]. Another quasi-experimental study, conducted in Chile during SFP dissemination, showed a reduction in coercive and permissive parenting practices such as yelling, insults, and lack of control when their children misbehave [24].

*Famílias Fortes* has also been shown to be effective in increasing nonviolent discipline education practices, with participating caregivers reporting increasing discipline strategies involving positive parenting practices. When children misbehave, caregivers explain why the child’s behavior is inappropriate and use non-aggressive parenting techniques like grounding, giving them something else to do, or even forbidding them to do something they like without any violent action [40]. Some program activities teach these techniques to parents and legal guardians by encouraging nonviolent educational strategies in response to inappropriate behavior, for example, when a child refuses to take a bath the consequence should be a reduction in video game time, not a spanking. Nonviolent discipline is a predictor of healthier youth development and less involvement in risky behaviors [52]. Similarly, a previous study evaluating the effect of SFP in the United States found that the program decreased aggressive and hostile behaviors in family interactions [22]. In another US study, the SFP applied in schools reduced adolescents’ hostility in family conflicts, but not the parents’ [25]. However, even though the program expanded the use of nonviolent educational practices, it failed to be effective in reducing verbal and physical violence. Such null fundings may be explained by the fact that the program activities put more emphasis on skills that should be acquired and not on those that need to be erased. Perhaps this is why the program was effective in increasing nonviolent discipline, but not in decreasing violent parental practices. In this regard, revisions should be made to the manual to insert clear messages of non-violence as an educational practice.

*Famílias Fortes* also showed an effect on caregivers’ drinking behavior, as they became less intoxicated in the presence of their children. Child exposure to increased parental drinking levels and a liberal attitude toward alcohol use are considered risk behaviors for alcohol use [53] and binge drinking [54] in adolescents. A Brazilian study observed a threefold increase in the odds of adolescent early alcohol consumption compared with those who had no experience with alcohol in the family [55].

Conversely, we observed no effect of the program in reducing the prevalence of lifetime drug use or in increasing risk perception about these substances. However, two aspects must be considered when analyzing these null findings: (1) this study evaluates short-term effects of the program (6-month follow-up after baseline collection); (2) we are dealing with adolescents who started the study with a mean age of 12.6 years and, 6 months later, were turning 13. Thus, two hypotheses should be considered. The first, and more robust, stems from the program’s logic model which understands the reduction in drug use as a distal outcome arising from changes in parenting styles, taken as proximal outcomes [14, 33]. Previous studies on program effect have shown a more consistent reduction in alcohol and drug use prevalence and initiation, with more significant results after 6 months of follow-up. A US study [14] followed students from 6th to 12th grade and showed a universal prevention shield effect. No significant difference between controls and intervention on drug use was observed at 6-month and 1-year follow-up; however at 2-, 3-, and 4-year follow-ups, the control group was more susceptible to initiation of illicit substance use [13]. A second hypothesis is that since Brazilian adolescents start consuming alcohol on average around 13.5 years old [38], our sample showed low prevalence of consumption of all substances. As such, age need to advance so adolescents can draw on the developed skills when the opportunity for experimentation arises. Consequently, new program evaluations with at least 12-month follow-up is required to effectively verify if any positive effect on drug use can occur.

Importantly, in European countries who implemented the same program as ours, it has failed to reproduce the same strong effects as the US version of reducing illicit drug use initiation by almost 50% in a 6-year follow-up [14], showing mostly null effects on all substance use outcomes. In Poland [19] and the UK [20], the program was not effective in reducing drug use at up to 2 years of follow-up; and in Sweden [18], at up to 4 years of follow-up. In contrast, a 50% reduction in the prevalence of cigarette experimentation was identified in Germany 18 months after program implementation [15]. In Spain, the program showed a reduction trend in drug use progression; however, the study had methodological limitations, as it did not use a gold standard for effectiveness evaluation [17].

Other hypotheses for this lack of effectiveness among adolescents include the implementers’ inability to carry out activities aimed at adolescents since these require more training than delivering sessions to adults, or even that the activities designed for adolescents might not be effective in reducing consumption since the program targets a very wide age range (adolescents from 10 to 14 years old). An activity designed for a 10-year-old might not engage a 14-year-old and vice versa. Another point to consider is the superficial or poorly conducted discussion about alcohol and other drugs in adolescent workshops, which, although present, may not have been sufficient or well conducted. Finally, even after a few years of follow-up, we may find that the program is not effective in reducing alcohol and drug use among adolescents, as identified in Europe.

As for study limitations, we highlight that 12% of the families were lost during the 6-month follow-up and, additionally, 23.3% of the families in the intervention group did not participate in all program meetings. However, this is a low sample loss compared with another Brazilian study with a sample loss of 38% [56], and other SFP studies, where attrition rates ranged from 21% [21] to 44% [25] after 6 months. Regarding the difficulty in maintaining participant adherence throughout the intervention, in Spain only 65.38% regularly attended the main program sessions, a much lower family participation than in our study [17]. But the sessions were held in places of easy access to families and by known SARC professionals [21]. Another limitation was assessment performed by self-reported scales rather than observations of family interactions as in Semeniuk et al. [25], which may generate information bias. However, most SFP studies have conducted assessments using self-completed questionnaires due to lower costs and less need for rater training [56]. Our findings cannot be generalized to the Brazilian population. Importantly, this is the only study in Latin America that evaluated SFP by RCTs, a gold standard study design for intervention evaluation [57]. Moreover, the findings need to be interpreted with caution as the effect sizes found are small and confidence intervals for some of the estimations are quite large.

In conclusion, the Brazilian version of the SFP 10–14 program showed effectiveness in enhancing parenting skills and nonviolent educational practices, as well as reducing parental drinking behavior near their children. These family-level positive results demonstrate the program’s preventive and health-promoting potential, since evidence about the influence of family behaviors on adolescent development is conclusive. Future studies should conducted a long-term evaluation of the program effects to see if changes in family outcomes achieve the preventive results for adolescent drug use, as well as investigate why the program failed to modify other outcomes predicted in its logic model.

At this stage, the researchers suggest that the program be continued, given its positive effects on family outcomes and its importance for Brazilian public policies related to family environment, drugs and violence, but in conjunction with further studies aimed at improving it. Potential issues regarding day and time of implementation (weekdays and working hours are not the best moment for meetings), lack of good physical structure and human resources for program delivery, the broad age of adolescents involved (10 to 14 years old), and literacy of vulnerable parents should be considered in a new cultural adapted version.

## Data Availability

The datasets used and/or analysed during the current study are available from the corresponding author on reasonable request.

## Footnotes

1 Barueri/SP, Campina Grande/PB, Cascavel/CE, Carapicuíba/SP, Eusebio/CE, Fortaleza/CE, Ingá/PB, Nova Iguaçu/RJ, Pacatuba/CE, São Gonçalo do Amarante/CE, São José dos Campos/SP, Sinop/MT. Seven municipalities are located in the Northeast, Brazil’s poorest region. Among those, five are small cities, with populations ranging from 17,600 to 94,000 inhabitants and HDI ranging from 0.592 to 0.788. One of these municipalities is medium-sized with a population of 402,000 inhabitants and an HDI of 0.720, while the other is a major city with 2.6 million inhabitants and an HDI of 0.754. The other five municipalities are located in the southern and central-western regions of the country, are more urban, and have populations ranging from 146,000 to 823,000 inhabitants, with HDI ranging from 0.749 to 0.807.
